# DEVELOPMENT AND TESTING OF THE PC-MET (PERSON-CENTERED MEASURE EVALUATION TOOL)

**DOI:** 10.1093/geroni/igad104.0989

**Published:** 2023-12-21

**Authors:** Sheryl Zimmerman, Benjamin Mast, Cynthia Port, Kimberly VanHaitsma, Sam Fazio

**Affiliations:** University of North Carolina at Chapel Hill, Chapel Hill, North Carolina, United States; University of Louisville, Louisville, Kentucky, United States; Self-employed, Bloomington, Indiana, United States; Penn State University, State College, Pennsylvania, United States; Alzheimer’s Association, Chicago, Illinois, United States

## Abstract

Despite the importance of person-centered measurement -- and that numerous research instruments are now available to measure person-centeredness -- there are no tools to evaluate the extent to which existing research instruments themselves are developed consistent with principles of person-centeredness. The PC-MET (Person-Centered Measure Evaluation Tool) was developed to fill that gap. Based on literature and theory, the PC-MET assesses the process of instrument development and the content of the instrument itself based on six overarching criteria of person-centeredness: co-creation, accommodation, incorporation, biopsychosocial-cultural components, pragmatism, and systemic focus. The inter-rater reliability of the PC-MET was evaluated based on instruments recommended for use in relation to the Alzheimer’s Association 2018 Dementia Care Practice Recommendations (DCPR). Independent raters evaluated two measures within each DCPR area using the PC-MET. Agreement was excellent (kappas>0.81) across all PC-MET criteria with the exception of the criteria assessing biopsychosocial-cultural components (where agreement was moderate; kappa=0.42) and pragmatism (where agreement was substantial; kappa=0.68). Raters provided feedback on the PC-MET and agreed or strongly agreed that the tool is easy to understand and score; the six PC-MET criteria are relevant and complete; and the PC-MET could improve the person-centeredness of dementia measurement instruments. The PC-MET has broad potential use, including to critique the extent to which existing instruments are person-centered, to promote efforts to make existing measures more person-centered, and to inform the development of new person-centered measures. This work was supported by the NIA through a grant to the Alzheimer’s Association and by the LINC-AD Research Steering Committee.

